# Influence of a Deep Learning Noise Reduction on the CT Values, Image Noise and Characterization of Kidney and Ureter Stones

**DOI:** 10.3390/diagnostics12071627

**Published:** 2022-07-05

**Authors:** Andrea Steuwe, Birte Valentin, Oliver T. Bethge, Alexandra Ljimani, Günter Niegisch, Gerald Antoch, Joel Aissa

**Affiliations:** 1 Department of Diagnostic and Interventional Radiology, Medical Faculty, University Dusseldorf, D-40225 Dusseldorf, Germany; birte.valentin@med.uni-duesseldorf.de (B.V.); oliver.t.bethge@med.hhu.de (O.T.B.); alexandra.ljimani@med.uni-duesseldorf.de (A.L.); antoch@med.uni-duesseldorf.de (G.A.); joel.aissa@med.uni-duesseldorf.de (J.A.); 2 Department of Urology, Medical Faculty, University Dusseldorf, D-40225 Dusseldorf, Germany; guenter.niegisch@med.uni-duesseldorf.de

**Keywords:** deep-learning, computed tomography, renal and ureteral stones, denoising

## Abstract

Deep-learning (DL) noise reduction techniques in computed tomography (CT) are expected to reduce the image noise while maintaining the clinically relevant information in reduced dose acquisitions. This study aimed to assess the size, attenuation, and objective image quality of reno-ureteric stones denoised using DL-software in comparison to traditionally reconstructed low-dose abdominal CT-images and evaluated its clinical impact. In this institutional review-board-approved retrospective study, 45 patients with renal and/or ureteral stones were included. All patients had undergone abdominal CT between August 2019 and October 2019. CT-images were reconstructed using the following three methods: filtered back-projection, iterative reconstruction, and PixelShine (DL-software) with both sharp and soft kernels. Stone size, CT attenuation, and objective image quality (signal-to-noise ratio (SNR), contrast-to-noise ratio (CNR)) were evaluated and compared using Bonferroni-corrected Friedman tests. Objective image quality was measured in six regions-of-interest. Stone size ranged between 4.4 × 3.1–4.4 × 3.2 mm (sharp kernel) and 5.1 × 3.8–5.6 × 4.2 mm (soft kernel). Mean attenuation ranged between 704–717 Hounsfield Units (HU) (soft kernel) and 915–1047 HU (sharp kernel). Differences in measured stone sizes were ≤1.3 mm. DL-processed images resulted in significantly higher CNR and SNR values (*p <* 0.001) by decreasing image noise significantly (*p <* 0.001). DL-software significantly improved objective image quality while maintaining both correct stone size and CT-attenuation values. Therefore, the clinical impact of stone assessment in denoised image data sets remains unchanged. Through the relevant noise suppression, the software additionally offers the potential to further reduce radiation exposure.

## 1. Introduction

In patients with acute flank pain and suspected renal or ureteral stones, non-contrast-enhanced computed tomography (CT) is recommended for diagnosis [1]. CT also allows for the assessment the composition and size of potential urinary tract stones [2,3]. The attenuation of reno-ureteric stones can provide information on their composition or origin. Furthermore, the size of the stones can influence the treatment strategy [4]. Measurements of stone size and CT-attenuation can be performed in either soft tissue window or bone window, depending on the clinic’s standard operating procedure. There is still no general or international standard on how the measurements should be performed. Both methods are described in the literature [5], though they are known to provide different measurements. Usually, soft-tissue window settings tend to overestimate stone size, while bone window settings tend to slightly underestimate stone size [6]. Furthermore, the attenuation is influenced by the size of the measurement area (region of interest) and partial volume effects.

A major concern of CT imaging is the associated radiation exposure and the potential carcinogenic effects [7,8]. Therefore, it is important to reduce the radiation burden of the CT, while maintaining its diagnostic accuracy. Dose reduction in CT is threefold and relates to (a) hardware optimization, (b) protocol optimization, and (c) post-processing software, i.e., noise-reduction techniques. Noise-reduction techniques, such as iterative reconstruction or deep-learning-based post-processing software allow for the reduction in the required radiation exposure [8,9]. However, the underlying principle of these techniques is often kept confidential. The potential effect on image quality and quantitative image information needs to be evaluated thoroughly prior to clinical application.

One example of a deep-learning-based software for noise suppression is PixelShine (AlgoMedica, Sunnyvale, CA, USA). PixelShine was already evaluated in terms of objective image quality in different body regions, such as low-dose abdominal CT and whole-body low-dose CT [8,10]. However, the influence on small structures, such as the size and CT-attenuation values of reno-ureteric stones have not been evaluated so far. Therefore, the aim of this study was to assess whether and to what degree the post-processing software PixelShine influences attenuation measurements and stone size in patients that undergo low-dose abdominal CT and the degree to which the objective image quality is altered by the artificial intelligence (AI)-based technique.

## 2. Materials and Methods

### 2.1. Patient Cohort

This IRB-approved retrospective study included all patients that underwent low-dose abdominal CT in our department with radiological indication of suspected stone disease and were diagnosed with at least one stone in the urinary tract between 16 August 2019 and 13 October 2019.

### 2.2. CT Acquisition

CT examinations were performed on a Somatom Definition Flash CT scanner (Siemens Healthineers, Forchheim, Germany) without contrast-enhancement. The protocol was a dedicated low-dose protocol. In detail, patients were scanned in the prone position and scan coverage included the upper poles of the kidneys to the pelvic floor. Scan parameters were a tube potential of 100 kVp, reference tube-current time product 80 mAs, collimation 128 × 0.6 mm, pitch 0.6, and rotation time 0.5 sec. Dose parameters (CTDIvol and DLP) were documented to calculate the effective dose with the DLP to effective dose conversion factor of k = 0.0151 mSv/(mGycm) [11].

### 2.3. Image Reconstruction

Six reconstructions were obtained:

(a) B30f: Filtered back-projection with a B30f kernel for soft tissue presentation.
(b) B70f: Filtered back-projection with a B70f kernel for bone or lung presentation.
(c) I30f: Iterative reconstruction (SAFIRE (Siemens Healthineers, Forchheim, Germany)) with an I30f kernel for soft tissue presentation.
(d) I70f: Iterative reconstruction (SAFIRE (Siemens Healthineers, Forchheim, Germany)) with an I70f kernel for bone or lung presentation.
(e) P30f: PixelShine (AlgoMedica), version 1.2.104, using the reconstructed images of (a) with the parameters P214A8S.
(f) P70f: PixelShine (AlgoMedica), version 1.2.104, using the reconstructed images of (b) with the parameters PB14A4L2.

The reconstructions (e) and (f) were obtained by sending the filtered-back projections from (a) and (b), stored in our picture archive and communication system (PACS, Sectra Medical Systems, Linköping, Sweden) to the PixelShine server. Since PixelShine is a commercial software product, the algorithm is kept confidential. Post-processed images were returned to PACS for further evaluation.

### 2.4. Image Analysis

Image analysis was performed in ImageJ version 1.52p (National Institute of Health, Bethesda, ML, USA). Circular regions-of-interest (ROIs) with a radius of approximately 10 mm (area 314 mm^2^) were drawn in the liver, spleen, paravertebral muscle, fat, vertebral body and in the air outside the patient for all six reconstructions for all patients. The position of the ROI was identical in each image set. Measured parameters were ROI area size, CT value, standard deviation (noise), and minimum and maximum CT values.

Subsequently, signal to noise ratio (*SNR*) and contrast to noise ratio (*CNR*) were calculated according the following formulas:

(1) $$SNR=\frac{signal\;in\;ROI}{noise\;in\;ROI}$$

(2) $$CNR=\frac{signal\;in\;stone-signal\;in\;fat}{noise\;in\;fat}$$

Furthermore, one radiologist-in-training with 4 years of experience (B.V.) in reading abdominal CT measured the size (x- and y-diameter) and CT-attenuation of all detected stones in all six image data sets for all patients. Stones were magnified for the measurement in order to obtain exact measurement boundaries. Reading of images was performed in PACS with anonymized image data (patient information and type of reconstruction were unknown to the radiologist). In case of multiple stones, the largest stone was evaluated. The same stone in each set of reconstructions was evaluated.

### 2.5. Analysis of Urinary Concrements

Analysis of urinary concrements was performed using Fourier-transform-infrared- (FT-IR) spectroscopy by an external accredited laboratory (Labor Limbach, Heidelberg, Germany) [12].

### 2.6. Statistical Analysis

Analysis of patient data was performed using Microsoft Excel 2016 (Redmond, WA, USA). Statistical differences in CT values, image noise and size measurements between the six reconstructions were calculated using SPSS version 28 (IBM, Chicago, IL, USA) [13]. For the statistical analyses, Friedman tests with related samples and post hoc Bonferroni-correction were performed. The level of significance was *p <* 0.05. Figures were built using R [14].

## 3. Results

### 3.1. Patient Characteristics

In the evaluated study period, 45 patients (32 males, 13 females) were diagnosed with a stone in the urinary tract. All stones were visible in each respective reconstruction. A mean CTDIvol of 2.6 ± 1.1 mGy (range 1.3–7.9 mGy) and a mean DLP of 108.9 ± 42.4 mGycm (range 47.4–288.5 mGycm) were obtained. The average effective dose amounted to 1.6 ± 0.6 mSv (range 0.7–4.4 mSv).

### 3.2. Stone Size

**Figure 1:**
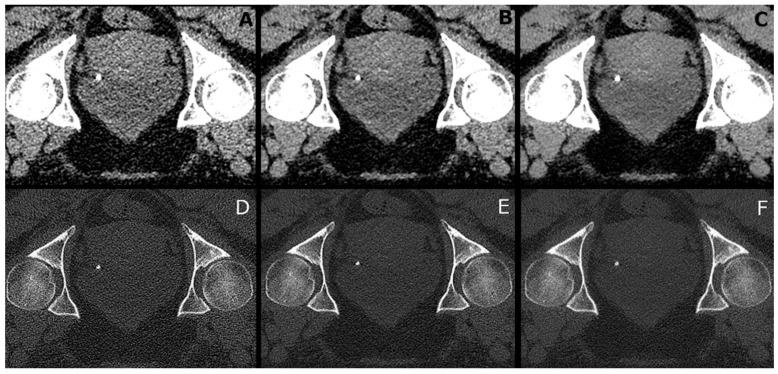
Image example of a patient with an ostial stone on the left side (prone position). (**A**–**C**): soft kernel, (**D**–**F**): sharp kernel: left: filtered back-projection, middle: iterative reconstruction, right: PixelShine applied on filtered back-projection. Soft tissue window: width 300 HU, level 40 HU. Bone window: width: 1500 HU, level 450 HU.

Stone size varied between measurements in soft tissue and sharp reconstructions. Stones in sharp kernel reconstructions presented with a more distinct edge than in soft kernel reconstructions. Differences are depicted in Figure 1.

In a direct comparison between soft tissue and sharp reconstructions, the measured stone sizes were significantly smaller (*p <* 0.001) when measured in sharp reconstructions, except for the x-diameter of the stones on iterative reconstructed images (*p* = 1.000). The largest differences in size amounted to 3.3 mm (P30f vs. P70f). Within one reconstruction kernel, differences in stone size were ≤1.3 mm. Results of statistical analyses are provided in Table 1.

When applying soft kernels, stone diameters (x- and y) measured with iterative reconstruction were smaller than measured on filtered back-projections or PixelShine-processed reconstructions in 87/90 (96.7%) and 83/90 (92.2%) of the cases, respectively. When applying sharp kernels, there were no statistically significant differences in the size measurements between the three reconstruction techniques.

### 3.3. CT-Attenuation Values of Stones

In a direct comparison between all reconstructions with sharp and soft kernels, the measured CT values were significantly higher in reconstructions with sharp kernels (*p <* 0.001).

**Figure 2:**
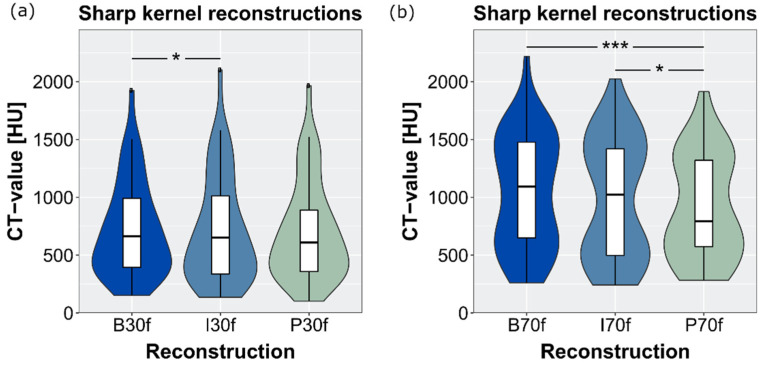
CT values measured from stones in (**a**) soft kernel reconstructions (filtered back-projection (B30f), iterative reconstruction (I30f) and PixelShine (P30f)) and (**b**) sharp kernel reconstructions ((filtered back-projection (B70f), iterative reconstruction (I70f) and PixelShine (P70f)). * *p* < 0.050, *** *p* < 0.001.

Mean CT values of stones in sharp and soft kernels are provided in Table 2, together with their statistical analysis. In general, attenuation measurements were lowest in PixelShine-processed images. Figure 2 visualizes the measured stone attenuations and their distributions. In one tiny stone (size <1 mm × 1 mm), the CT value in the B70f-reconstruction was 751 HU higher than in P70f- and I70f-reconstructions.

### 3.4. Stone Composition

A urinary stone analysis was available for 25/45 patients (55.6%). The majority of stones were composed of calcium-oxalate (15/45, 33.3%). Other stones were composed of a mixture of calcium-oxalate and carbonate apatite (5/45, 11.1%), or uric acid (3/45, 6.7%), of a mixture of carbon apatite and magnesium ammonium phosphate (1/45, 2.2%) and of cysteine (1/45%, 2.2%). Corresponding CT values are presented in Table 3. Although the CT values of stones composed of uric acid and calcium oxalate are similar for soft kernel reconstructions, differences are larger for sharp kernel reconstructions.

### 3.5. CT Values and Image Noise in Tissues and Air

Differences in CT values between ROIs in liver, spleen, fat, and muscle were comparable in reconstructions with soft kernels, with maximum differences of 0.5% between the reconstructions (see Table 4a). In air and bone ROIs reconstructed with sharp kernels, differences in attenuation were within 5.5%. Still, there were significant differences in mean attenuation.

Image noise varied between the reconstructions as follows (see Table 4b): highest noise values were measured in filtered back-projections whereas lowest noise values were measured with the denoising software PixelShine. Differences between the reconstructions were significant (*p <* 0.001). In fat, image noise was 57% lower in P30f- than in B30f-reconstructions.

### 3.6. SNR and CNR

**Figure 3:**
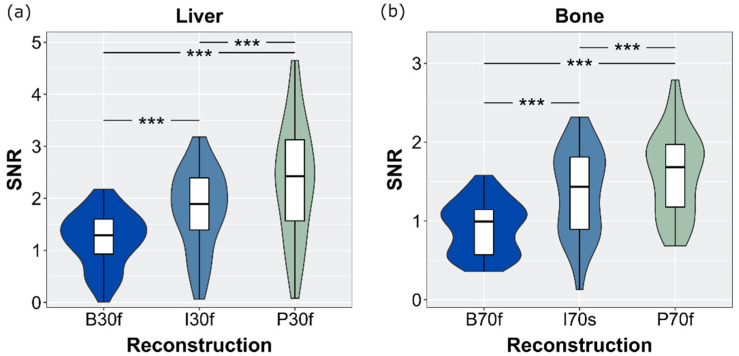
Signal to noise ratio calculated in the liver (**a**) and bone (**b**). Differences between the three reconstruction techniques were significant (*** *p* < 0.001).

The highest SNR values independent of the kernel type and tissue were determined for PS-reconstructed images. Due to higher noise values when applying sharp kernels, SNR values were considerably lower compared to applying soft kernels, where the image noise was suppressed. See Figure 3 for the SNR measured in the liver and bone.

**Figure 4:**
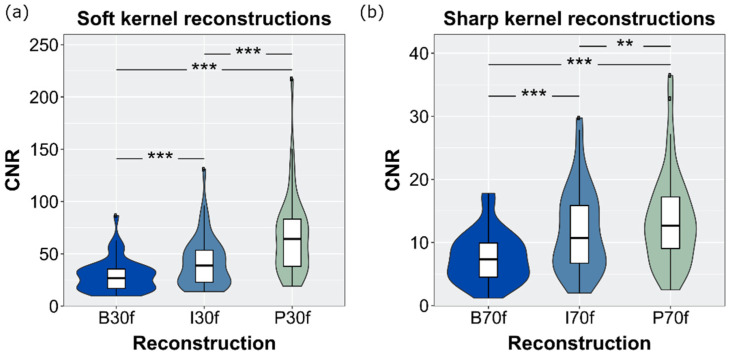
Contrast to noise ratio for (**a**) soft kernel and (**b**) sharp kernel reconstructions. Differences between the three reconstruction techniques were significant (*** *p* < 0.001, ** *p* = 0.002).

Highest CNR values measured in stones and fat tissue were determined for PixelShine due to lowest noise levels both in soft tissue and in bone reconstructions (CNR 29.3 for B30f compared to 67.1 for P30f). Differences between the kernels were statistically significant (*p <* 0.001) (see Figure 4).

## 4. Discussion

Reno-ureteric stones were evaluable with traditional filtered back-projection, iterative reconstruction, and the novel deep-learning method PixelShine. Sharp kernel reconstructions resulted in smaller stone size measurements and significantly higher CT-attenuation values than soft kernel reconstructions. The differentiation between stone compositions was improved using sharp kernel reconstructions. Using AI-based methods offers increased signal-to-noise and contrast-to-noise ratios with the potential to further reduce radiation exposure to the patient.

There is still no gold standard for how to measure the attenuation and size of stones, e.g., with a defined reconstruction method or type of kernel. Especially with the increasing number of scanner-integrated or vendor-independent post-processing techniques, the comparability between the different methods may be difficult.

The software PixelShine has already been evaluated in a few technical and clinical research investigations, such as in ultra-low dose abdominal, pelvic, or midfacial trauma CT [8,10,15,16,17,18,19]. The publications show that the deep learning technique provides diagnostic images even at radiation exposures of 30% of the initial dose, regardless of the scanner type or reconstruction technique [8]. This is achieved by vigorously reducing image noise, resulting in increased signal-to-noise and contrast-to-noise ratios [8,10,15,18]. However, until now, no evaluation of (a) the detection and (b) the characterization of reno-ureteric stones in PixelShine-post-processed low-dose computed tomography of the abdomen has been published. The influence of deep learning techniques on the detection, image quality, size and attenuation of stones has already been studied for other vendors (AiCE, GE Healthcare and TrueFidelity^TM^_,_ Canon Medical Solutions) (see Table 5) [20,21,22]. Comparable to our results, the aforementioned techniques reduce noise, possibly allowing for a reduction in the radiation dose in the future. Unfortunately, smaller stones <3mm could possibly be missed when reducing the radiation exposure, so the techniques should still be considered with caution.

**Figure 5:**
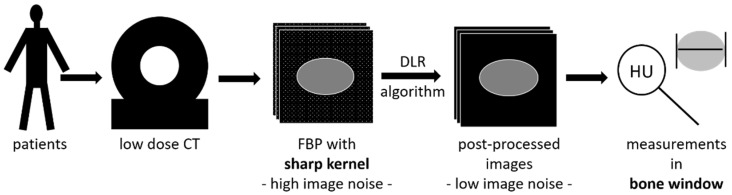
Proposed method for diagnosis of reno-ureteric stones: Patients should undergo low-dose computed tomography (CT), reconstructed with filtered back-projection (FBP) with a sharp kernel and post-processed with PixelShine as deep learning reconstruction (DLR) algorithm to remove image noise. All measurements (size, CT attenuation in Hounsfield units (HU)) should be performed in bone window settings.

In general, reno-ureteric stones were evaluable in the post-processed reconstructions in this study, both with a sharp kernel and a soft kernel. No stone was missed. However, CT values and stone sizes differed between the three evaluated techniques. When measuring the CT values in the six evaluated image data sets per patient, the highest CT numbers were determined using sharp kernel and filtered back-projection as reconstruction methods. Iterative reconstruction and PixelShine resulted in lower CT-attenuation values. The measured stone diameters explain the difference. Diameters in reconstructions with sharp kernels were approximately 1 mm smaller compared to the diameters in soft kernel reconstructions. Presuming that the stone size is smaller since its margins are better distinguishable when employing sharp kernels, the CT value is more likely to be measured in the centre region of the stone. In this case, the periphery of the stone that might already contain soft tissue instead of stone material is elided. Therefore, less partial volume artifacts influence the measurement of attenuation, which would decrease the average CT value. Therefore, we recommend the diagnosis and measurement of reno-ureteric stones using sharp reconstructions and bone window settings (see Figure 5).

Lidén et al. evaluated the impact of image post-processing parameters on the size of renal stones and further assessed the inter- and intra-reader variability of stone size measurements [23]. They noticed considerable differences between reconstructions of different slice thickness and increment, window settings (bone vs. soft tissue), and furthermore, an intra-reader variability of ±0.5 mm and an inter-reader variability of ±1.3 mm. They concluded that a difference in size estimation of a structure of one or two millimeters is of no significance in most clinical situations [23]. In our study, differences in sizes were of a maximum 3.3 mm between soft and sharp kernel reconstructions and 1.3 mm within one reconstruction kernel. These differences are likely to have no significance on the treatment of patients.

Although differences in CT values of the stones were visible within one kernel, the CT value only influenced the immediate treatment if the stone passed without medical intervention. Unfortunately, single-source CT cannot provide information on the stone composition that is as detailed as that of a dual-energy CT [24,25,26]. It is possible to obtain a rough differentiation between different compositions by means of the CT value; however, there is no strict cut-off value for each composition (e.g., uric acid, cysteine, calcium) [27]. In general, stones consisting of uric acid have CT values of lower than 600 HU, which was supported by our study [2,3,28,29]. Stones consisting of calcium oxalate tend to have higher CT values (>1000 HU), again supported by our study [2,3,28,29]. We were able to see that the differentiation between stones composed of uric acid and stones composed of calcium oxalate is clearer with sharp kernel reconstructions. In the case of medical intervention due to an immobile stone, differentiation helps to induce a medicinal therapy for uric acid stones, whereas calcium oxalate stones require interventional therapy.

In this study, the range of CT values within one reconstruction kernel could result in a misinterpretation of the composition since differences in CT values could be as large as 750 HU; however, this is only the situation in rare cases and with very small stones. The referring doctor usually only receives information regarding the occurrence, size and position of a stone rather than the composition. The direct treatment is usually based on the clinical presentation and patient symptoms rather than the stone composition. Furthermore, stones are frequently calcified, which might hide the actual stone composition [30].

Unfortunately, many articles do not describe the reconstruction kernel they use, rather only detailing the window settings (e.g., bone or soft tissue window). However, the reconstruction kernel changes the sharpness an image noise of structures [31]. Typically, soft tissue reconstruction kernels tend to smoothen tissue edges. In contrast, sharp kernels are often edge enhancing, creating sharper and more distinct tissue edges. Sharp kernels influence the size of a small structures and consequently, also the CT value of measured stones, since the partial volume effects are reduced. Nevertheless, even when using the same kernel, CT values and stone sizes are influenced by employing different window settings (bone vs. soft tissue windows) [23,28,29,32].

Both soft and sharp reconstruction kernels exhibit advantages and disadvantages in the detection and measurement of stones. The anatomical classification is easier with soft kernels and soft tissue window, as the ureters and their anatomical course can be better distinguished here. However, the detectability of stones is usually higher when using sharp kernels and bone window, since the high attenuation of stones in these settings is more prominent compared to soft tissue settings. Umbach et al. and Danilovic et al. proposed using bone window and small slice thickness to determine the stone size due to a higher accuracy compared to measurements in soft tissue window settings [32,33]. However, they did not provide information on the employed reconstruction filter. Moreover, Eisner et al. also proposed using bone window settings and to magnify the image to increase the accuracy of stone measurements [34].

Independent of the stone characteristics, this study also examined the objective image quality between the six different reconstructions. PixelShine’s image noise reduction ability was already proven in different studies [8,10,15,18]. This study demonstrated the highest signal-to-noise and contrast-to-noise ratios with reconstructions employing PixelShine, both for soft and sharp kernels. A noise reduction in the region around the stones might improve the detection of very small stones. However, in this study, all stones could be detected in all reconstructions, independent of the noise level. Some limitations need to be mentioned. The evaluation of stones in terms of material compositions is best performed by means of dual-energy CT [35]. However, this technique is not always available in every center and often is associated with higher radiation exposure [24,35,36]. In our institute, the detection of reno-ureteric stones was performed with a single tube potential. Furthermore, stone size was not available for any of the patients, and composition was only available for some patients. Hence, we could not evaluate stone composition and stone size thoroughly. However, this study did not aim at the exact stone composition. Furthermore, other reconstruction methods, such as ADMIRE (Siemens Healthcare, Forchheim, Germany) might have resulted in different characterizations. Additionally, since the software PixelShine is a commercial product, the exact algorithm is kept confidential and it can be used simply to evaluate the results.

## 5. Conclusions

The size and CT value of reno-ureteric stones differ between sharp and soft reconstruction kernels. Within one type of kernel, post-processing methods such as PixelShine influence the measurements to a certain degree, however, this does not impede the clinical decision. Currently, the software is not part of our standard reconstruction process, but is only employed for research purposes. Therefore, PixelShine images were reconstructed in a subsequent step. However, it is possible to integrate the reconstruction process in the general workflow. A noise-reduction algorithm, which can decrease the radiation exposure of these patients is of great advantage and is highly recommended.

Yet, a standard measuring procedure within one institute is required, since the differences in size and CT value between soft and sharp kernel reconstructions were statistically significant and so might be useful for further treatment. In general, we recommend the usage of PixelShine and sharp kernel reconstructions, diagnosed in a bone window to increase the differentiability of stone compositions.

## Figures and Tables

**Table 1:** Results of the stone size measurements and statistical analysis. Size measurements of the three evaluated reconstructions were compared (B vs. I, I vs. P, P vs. B). Differences between measurements with shared superscripts were statistically significant. There were no significant differences between sharp kernel reconstructions.

	x (mm)	y (mm)	x (mm)	y (mm)
	Soft Kernels		Sharp Kernels	
B	5.6 ± 3.0 ^AC^	4.2 ± 2.2 ^E^	4.4 ± 3.0	3.1 ± 2.0
I	5.1 ± 3.1 ^AB^	3.8 ± 2.1 ^DE^	4.4 ± 3.0	3.1 ± 2.1
P	5.4 ± 3.1 ^BC^	4.1 ± 2.2 ^D^	4.4 ± 3.0	3.2 ± 2.1

Results of the statistical comparisons: A, B, D, E: *p <* 0.001; C: *p =* 0.006. Abbreviations: B: filtered back-projection, I: iterative reconstruction; P: PixelShine.

**Table 2:** Results of the stone CT-value measurements and statistical analysis. Stone CT values of the three evaluated reconstructions were compared (B vs. I, I vs. P, P vs. B). Differences between CT values with shared superscripts were statistically significant.

	Soft Kernels	Sharp Kernels
B	717.6 ± 405.9 ^A^	1047.3 ± 490.7 ^C^
I	714.4 ± 459.4 ^A^	986.2 ± 516.5 ^B^
P	704.2 ± 424.5	915.9 ± 449.6 ^BC^

Results of the statistical comparisons: A: *p* = 0.046, B: *p* = 0.040, C: *p* < 0.001. Abbreviations: B: filtered back-projection, I: iterative reconstruction; P: PixelShine.

**Table 3:** Composition of the stones and corresponding attenuation values where results of X-ray diffraction were available. Data provided as median with 25%- and 75%-quartiles in parentheses, where *n* > 1 stone was available.

		Soft Kernel Reconstruction			Sharp Kernel Reconstruction		
Composition	*n*	B	I	P	B	I	P
CaOx	15	772 (523–1059)	739 (508–1147)	829 (531–1044)	1316 (1045–1583)	1251 (703–1565)	1119 (791–1399)
Calcium-Oxalate-carbonate apatite	5	703 (663–777)	672 (668–675)	632 (598–699)	1092 (1000–1339)	1251 (1156–1254)	850 (764–1124)
Uric acid	3	501 (430–664)	513 (412–645)	515 (422–562)	460 (452–967)	464 (434–1040)	507 (463–972)
Cystine	1	721	714	725	731	745	714
Carbonate-Apatite-mix	1	1174	1257	1202	1149	1358	1320

**Table 4:** Results of the CT value measurements and statistical analysis in the regions-of-interest liver, spleen, muscle, fat (soft kernels) and in the air and lung (sharp kernels). CT values of the three evaluated reconstructions were compared (B vs. I, I vs. P, P vs. B). Differences between CT values with shared superscripts were statistically significant.

**(a)**			
**CT Value**	**B**	**I**	**P**
Air (sharp)	−940.0 ± 12.0 ^A^	−933.4 ± 11.2 ^AB^	−939.4 ± 11.9 ^B^
Bone (sharp)	188.8 ± 63.3 ^D^	186.0 ± 66.7 ^C^	196.2 ± 63.5 ^CD^
Liver (soft)	44.7 ± 16.3	44.7 ± 16.1	44.6 ± 16.1
Muscle (soft)	51.3 ± 6.7 ^EF^	51.1 ± 6.7 ^F^	50.8 ± 7.0 ^E^
Spleen (soft)	45.4 ± 3.5	45.4 ± 3.4	45.4 ± 3.4
Fat (soft)	−116.1 ± 9.7 ^H^	−116. ± 9.6 ^G^	−115.6 ± 9.6 ^GH^
Results of the statistical comparisons: A–G: *p* < 0.001, H: *p* = 0.001.			
**(b)**			
**Image Noise**	**B**	**I**	**P**
Air (sharp)	77.5 ± 15.9	49.4 ± 13.5	28.5 ± 14.3
Bone (sharp)	212.3 ± 40.1	143.0 ± 31.1	124.0 ± 20.7
Liver (soft)	38.2 ± 7.7	26.2 ± 5.0	20.3 ± 4.8
Muscle (soft)	31.6 ± 5.6	21.7 ± 3.9	14.5 ± 2.6
Spleen (soft)	34.5 ± 7.0	23.4 ± 4.8	16.4 ± 4.1
Fat (soft)	29.4 ± 5.6	20.4 ± 4.2	12.8 ± 3.1
Statistical differences (*p <* 0.001) among all reconstructions within one ROI. Abbreviations: B: filtered back-projection, I: iterative reconstruction; P: PixelShine.			

**Table 5:** Comparison of deep-learning based reconstruction tools employed for the diagnosis of kidney and ureter stones.

Parameter	This Study		Zhang et al. [20]		Thapaliya et al. [21]		Delabie et al. [22]	
Vendor	Algomedica	Siemens Healthineers	Canon Medical Systems		Canon Medical Systems		GE Healthcare	
Techniques used	PixelShine	IR (Safire), FBP	DLR (AiCE)	HIR	DLR (AiCE, six options evaluated)	AIDR3D	DLR (TrueFidelity^TM^)	FBP ASiR-V
Preprocessing techniques	FBP	Raw data	None described	Raw data	Raw data		None described	
Type of dataset used	CT of kidney stones in 45 patients, both soft tissue and bone kernel and window settings		CT of kidney stones in 51 patients with intra-individual comparison, soft tissue window settings; LDCT-HIR as gold standard		CT of kidney stones in 7 patients, AIDR3D as gold standard, soft tissue window		CT of kidney stones in 75 patients, soft tissue window (stone detection), bone window (stone count)	
Evaluation measures	Image noise, CNR, SNR, attenuation, stone size		Radiation exposure, stone characteristics, image noise, SNR, subjective IQ		Stone detection, stone size, inter-rater reliability		Attenuation, noise measurements, SNR, contrast, CNR, detectability, IQ, stone size category;	
Advantage	Higher objective IQ	Direct reconstruction	Reduced radiation exposure, higher IQ		High level of agreement with AIDR3D		Quantitative and qualitative IQ improved	
Disadvantage	Secondary reconstruction	Image noise	Lower sensibility	Higher sensibility		More image noise than AiCE	Contrast between kidney and spleen different to ASiR-V	Image noise
Recommendation	Usage of PixelShine to reduce image noise; use sharp kernel reconstructions bone window to improve differentiation between stone compositions		DLR with ultra-low dose CT to reduce dose, though it might miss stones <3mm		Usage of DLR to potentially reduce radiation exposure		Usage of DLR to improve IQ, though it still might miss stones <3mm	

Abbreviations: CT: computed tomography, CNR: contrast-to-noise ratio, DLR: Deep-learning reconstruction, HIR: hybrid iterative reconstruction, IQ: image quality, LDCT: low-dose CT, SNR: signal-to-noise-ratio.

## Data Availability

Data are contained within the article.

## References

[1] Nambiar A.K., Bosch R., Cruz F., Lemack G.E., Thiruchelvam N., Tubaro A., Bedretdinova D.A., Ambühl D., Farag F., Lombardo R. (2018). EAU Guidelines on Assessment and Nonsurgical Management of Urinary Incontinence. Eur. Urol..

[2] Kawahara T., Miyamoto H., Ito H., Terao H., Kakizoe M., Kato Y., Ishiguro H., Uemura H., Yao M., Matsuzaki J. (2016). Predicting the Mineral Composition of Ureteral Stone Using Non-Contrast Computed Tomography. Urolithiasis.

[3] Shahnani P.S., Karami M., Astane B., Janghorbani M. (2014). The comparative survey of Hounsfield units of stone composition in urolithiasis patients. J. Res. Med. Sci..

[4] Andrabi Y., Patino M., Das C.J., Eisner B., Sahani D.V., Kambadakone A. (2015). Advances in CT Imaging for Urolithiasis. Indian J. Urol..

[5] Coll D.M., Varanelli M.J., Smith R.C. (2002). Relationship of Spontaneous Passage of Ureteral Calculi to Stone Size and Location as Revealed by Unenhanced Helical CT. AJR Am. J. Roentgenol..

[6] Argüelles Salido E., Aguilar García J., Lozano-Blasco J.M., Subirá Rios J., Beardo Villar P., Campoy-Martínez P., Medina-López R.A. (2013). Lithiasis Size Estimation Variability Depending on Image Technical Methodology. Urolithiasis.

[7] Brenner D.J., Hall E.J. (2007). Computed Tomography—An Increasing Source of Radiation Exposure. N. Eng. J. Med..

[8] Brendlin A.S., Plajer D., Chaika M., Wrazidlo R., Estler A., Tsiflikas I., Artzner C.P., Afat S., Bongers M.N. (2022). AI Denoising Significantly Improves Image Quality in Whole-Body Low-Dose Computed Tomography Staging. Diagnostics.

[9] Yeoh H., Hong S.H., Ahn C., Choi J.-Y., Chae H.-D., Yoo H.J., Kim J.H. (2021). Deep Learning Algorithm for Simultaneous Noise Reduction and Edge Sharpening in Low-Dose CT Images: A Pilot Study Using Lumbar Spine CT. Korean J. Radiol..

[10] Steuwe A., Weber M., Bethge O.T., Rademacher C., Boschheidgen M., Sawicki L.M., Antoch G., Aissa J. (2021). Influence of a novel deep-learning based reconstruction software on the objective and subjective image quality in low-dose abdominal computed tomography. Br. J. Radiol..

[11] Deak P.D., Smal Y., Kalender W.A. (2010). Multisection CT Protocols: Sex- and Age-Specific Conversion Factors Used to Determine Effective Dose from Dose-Length Product. Radiology.

[12] Franck P., Nabet P., Dousset B. (1998). Applications of Infrared Spectroscopy to Medical Biology. Cell. Mol. Biol..

[13] Patil I. (2021). Visualizations with Statistical Details: The “ggstatsplot” Approach. J. Open Source Softw..

[14] R Core Team R: A Language and Environment for Statistical Computing. https://www.r-project.org/.

[15] Tian S., Liu A., Liu J., Liu Y., Pan J. (2019). Potential Value of the PixelShine Deep Learning Algorithm for Increasing Quality of 70 KVp+ASiR-V Reconstruction Pelvic Arterial Phase CT Images. Jpn. J. Radiol..

[16] Rozema R., Kruitbosch H.T., van Minnen B., Dorgelo B., Kraeima J., van Ooijen P.M.A. (2021). Iterative Reconstruction and Deep Learning Algorithms for Enabling Low-Dose Computed Tomography in Midfacial Trauma. Oral Surg. Oral Med. Oral Pathol. Oral Radiol..

[17] Rozema R., Kruitbosch H.T., van Minnen B., Dorgelo B., Kraeima J., van Ooijen P.M.A. (2022). Structural Similarity Analysis of Midfacial Fractures—a Feasibility Study. Quant. Imaging Med. Surg..

[18] Hasegawa A., Ishihara T., Thomas M.A., Pan T. (2022). Noise Reduction Profile: A New Method for Evaluation of Noise Reduction Techniques in CT. Med. Phys..

[19] Pan T., Hasegawa A., Luo D., Wu C.C., Vikram R. (2020). Technical Note: Impact on Central Frequency and Noise Magnitude Ratios by Advanced CT Image Reconstruction Techniques. Med. Phys..

[20] Zhang G., Zhang X., Xu L., Bai X., Jin R., Xu M., Yan J., Jin Z., Sun H. (2022). Value of Deep Learning Reconstruction at Ultra-Low-Dose CT for Evaluation of Urolithiasis. Eur. Radiol..

[21] Thapaliya S., Brady S.L., Somasundaram E., Anton C.G., Coley B.D., Towbin A.J., Zhang B., Dillman J.R., Trout A.T. (2022). Detection of Urinary Tract Calculi on CT Images Reconstructed with Deep Learning Algorithms. Abdom. Radiol..

[22] Delabie A., Bouzerar R., Pichois R., Desdoit X., Vial J., Renard C. (2021). Diagnostic Performance and Image Quality of Deep Learning Image Reconstruction (DLIR) on Unenhanced Low-Dose Abdominal CT for Urolithiasis. Acta Radiol..

[23] Lidén M., Andersson T., Geijer H. (2011). Making Renal Stones Change Size—Impact of CT Image Post Processing and Reader Variability. Eur. Radiol..

[24] Appel E., Thomas C., Steuwe A., Schaarschmidt B.M., Brook O.R., Aissa J., Hennenlotter J., Antoch G., Boos J. (2021). Evaluation of Split-Filter Dual-Energy CT for Characterization of Urinary Stones. Br. J. Radiol..

[25] Lazar M., Ringl H., Baltzer P., Toth D., Seitz C., Krauss B., Unger E., Polanec S., Tamandl D., Herold C.J. (2020). Protocol Analysis of Dual-Energy CT for Optimization of Kidney Stone Detection in Virtual Non-Contrast Reconstructions. Eur. Radiol..

[26] Bonatti M., Lombardo F., Zamboni G.A., Pernter P., Pycha A., Mucelli R.P., Bonatti G. (2017). Renal Stones Composition in Vivo Determination: Comparison between 100/Sn140 KV Dual-Energy CT and 120 KV Single-Energy CT. Urolithiasis.

[27] Sheir K.Z., Mansour O., Madbouly K., Elsobky E., Abdel-Khalek M. (2005). Determination of the Chemical Composition of Urinary Calculi by Noncontrast Spiral Computerized Tomography. Urol. Res..

[28] Gallioli A., de Lorenzis E., Boeri L., Delor M., Zanetti S.P., Longo F., Trinchieri A., Montanari E. (2017). Clinical Utility of Computed Tomography Hounsfield Characterization for Percutaneous Nephrolithotomy: A Cross-Sectional Study. BMC Urol..

[29] Torricelli F.C.M., Marchini G.S., De S., Yamaçake K.G.R., Mazzucchi E., Monga M. (2014). Predicting Urinary Stone Composition Based on Single-Energy Noncontrast Computed Tomography: The Challenge of Cystine. Urology.

[30] Thomas C., Heuschmid M., Schilling D., Ketelsen D., Tsiflikas I., Stenzl A., Claussen C.D., Schlemmer H.-P. (2010). Urinary Calculi Composed of Uric Acid, Cystine, and Mineral Salts: Differentiation with Dual-Energy CT at a Radiation Dose Comparable to That of Intravenous Pyelography. Radiology.

[31] Mackin D., Ger R., Gay S., Dodge C., Zhang L., Yang J., Jones A.K., Court L. (2019). Matching and Homogenizing Convolution Kernels for Quantitative Studies in Computed Tomography. Investig. Radiol..

[32] Danilovic A., Rocha B.A., Marchini G.S., Traxer O., Batagello C., Vicentini F.C., Torricelli F.C.M., Srougi M., Nahas W.C., Mazzucchi E. (2019). Computed Tomography Window Affects Kidney Stones Measurements. Int. Braz. J. Urol..

[33] Umbach R., Müller J.-K., Wendt-Nordahl G., Knoll T., Jessen J.P. (2019). In-Vitro Comparison of Different Slice Thicknesses and Kernel Settings for Measurement of Urinary Stone Size by Computed Tomography. Urolithiasis.

[34] Eisner B.H., Kambadakone A., Monga M., Anderson J.K., Thoreson A.A., Lee H., Dretler S.P., Sahani D.v. (2009). Computerized Tomography Magnified Bone Windows Are Superior to Standard Soft Tissue Windows for Accurate Measurement of Stone Size: An In Vitro and Clinical Study. J. Urol..

[35] Apfaltrer G., Dutschke A., Baltzer P.A.T., Schestak C., Özsoy M., Seitz C., Veser J., Petter E., Helbich T.H., Ringl H. (2020). Substantial Radiation Dose Reduction with Consistent Image Quality Using a Novel Low-Dose Stone Composition Protocol. World J. Urol..

[36] Lam J.P., Alexander L.F., William H.E., Hodge D.O., Kofler J.M., Morin R.L., Thiel D.D., Cernigliaro J.G. (2021). In Vivo Comparison of Radiation Exposure in Third-Generation vs Second-Generation Dual-Source Dual-Energy CT for Imaging Urinary Calculi. J. Endourol..

